# The Modern Dance as It Affects Eugenics

**Published:** 1913-06

**Authors:** 


### The Modern Dance as it Affects Eugenics.

In all ages and among all races and civilizations dancing has been, and is now intimately related to the sexual life. The psychology of this is not far to find. Rhythmical movements is a stimulant to tumescence, which, uncontrolled, excites the sexual feeling. With many tribes dancing is the mere prelude to sexual indulgence, and every civilization of the past was sullied by the licentiousness and wanton abandonment of the dance.

Within the influence of modern history polite society has sought to hide the motive that inspires the dance, and to see in its rhythm and cadence the opportunity to regulate physical energy. It is a means of escape to pent up passions. Perhaps, unconsciously indulged in by many, and may be regarded as a refinement in the conduct of social entertainments. That sexual impulse is the true motive of the dance is attested by the favor with which the "ragtime" variety is received in preference over the stately and genteel minuet type. The swing and action (not the rhythm and cadence) of the "ragtime" affords just the stimulus desired, and the opportunity is taken to indulge the feelings with as much show to decency as possible. In the name of polite society this is permitted as being in good form. But if such preference is encouraged how long will it be before this thin veneer of deception is punctured and the true motive revealed? May we not ask as the next step, Is there not imperative danger of the old debauchery and orgies of the past being re-enacted?

The highest moral type is the basis of Eugenics, so the upholding of the moral standard is the concern of this new religion. Therefore, Eugenics appeals to the parenthood of the country, to the leaders of society and the influences that control to stay the hand of this veritable vampire, ere it blights forever the "spark of life" that must be held inviolate if future generations are to exist.

